# Long Bone Structure and Strength Depend on BMP2 from Osteoblasts and Osteocytes, but Not Vascular Endothelial Cells

**DOI:** 10.1371/journal.pone.0096862

**Published:** 2014-05-16

**Authors:** Sarah H. McBride, Jennifer A. McKenzie, Bronwyn S. Bedrick, Paige Kuhlmann, Jill D. Pasteris, Vicki Rosen, Matthew J. Silva

**Affiliations:** 1 Department of Orthopaedic Surgery, Musculoskeletal Research Center, Washington University in St. Louis, St. Louis, Missouri, United States of America; 2 Department of Orthopaedic Surgery, Saint Louis University, St. Louis, Missouri, United States of America; 3 Department of Earth and Planetary Sciences, Washington University in St. Louis, St. Louis, Missouri, United States of America; 4 Department of Developmental Biology, Harvard School of Dental Medicine, Boston, Massachusetts, United States of America; University of Sheffield, United Kingdom

## Abstract

The importance of bone morphogenetic protein 2 (BMP2) in the skeleton is well known. BMP2 is expressed in a variety of tissues during development, growth and healing. In this study we sought to better identify the role of tissue-specific BMP2 during post-natal growth and to determine if BMP2 knockout affects the ability of terminally differentiated cells to create high quality bone material. We targeted BMP2 knockout to two differentiated cell types known to express BMP2 during growth and healing, early-stage osteoblasts and their progeny (osterix promoted Cre) and vascular endothelial cells (vascular-endothelial-cadherin promoted Cre). Our objectives were to assess post-natal bone growth, structure and strength. We hypothesized that removal of BMP2 from osteogenic and vascular cells (separately) would result in smaller skeletons with inferior bone material properties. At 12 and 24 weeks of age the osteoblast knockout of BMP2 reduced body weight by 20%, but the vascular knockout had no effect. Analysis of bone in the tibia revealed reductions in cortical and cancellous bone size and volume in the osteoblast knockout, but not in the vascular endothelial knockout. Furthermore, forelimb strength testing revealed a 30% reduction in ultimate force at both 12 and 24 weeks in the osteoblast knockout of BMP2, but no change in the vascular endothelial knockout. Moreover, mechanical strength testing of femurs from osteoblast knockout mice demonstrated an increased Young’s modulus (greater than 35%) but decreased post-yield displacement (greater than 50%) at both 12 and 24 weeks of age. In summary, the osteoblast knockout of BMP2 reduced bone size and altered mechanical properties at the whole-bone and material levels. Osteoblast-derived BMP2 has an important role in post-natal skeletal growth, structure and strength, while vascular endothelial-derived BMP2 does not.

## Introduction

Bone morphogenetic protein 2 (BMP2) plays critical roles in the skeleton. Extensive research has focused on BMP2’s ability to enable differentiation of precursor cells into osteoblasts and enhance osteoblast function[1]–[5]. BMP2 is highly expressed in osteoblasts [6]–[8], but little is known about how a deficiency of BMP2 affects differentiated osteogenic cells [9]–[11]. Furthermore, research has indicated that BMP2 is expressed in a variety of non-osseous tissues during development, growth, and healing [3], [6], [12], [13]. It is unknown if a lack of BMP2 in these other tissue types affects post-natal bone growth.

*BMP2* null mice are embryonic lethal due to defects in heart development and mesoderm [14]. Thus, conditional knockout mice must be used to study the effect of BMP2 deficiency on bone development and growth [9], [10]. Tsuji and colleagues knocked out BMP2 in all osteo- and chondro-progenitor cells and their lineages (e.g. chondrocytes, osteoblasts and osteocytes) using the limb specific promoter Prx1-Cre [10]. Surprisingly, these knockout mice were similar to wildtype mice at birth. However, as early as one week postnatally they began to exhibit defects in both cartilage and bone formation, and as they matured the phenotype worsened. Knockout mice had thinner, less dense bones that spontaneously fractured. However, it is unclear if one cell type (progenitor, chondrocyte, osteoblast, osteocyte) is the major contributor of BMP2 causing the phenotype or if the phenotype is a result of knockout from all chondro- and osteo- cells. Also, it is unknown if the spontaneous fractures were due solely to the smaller size of the knockout bones or if there was a defect in the bone material of knockout mice [9], [10]. In support of the latter, a recent report indicates that BMP2 deletion in cells of the osteoblast lineage results in a brittle bone phenotype [15].

Bone development, growth, remodeling, and healing are dependent on vascular cells and angiogenesis. For example, mineralization of the cartilaginous template follows vascular invasion [16]. Also, the periosteum, the bilayer membrane surrounding bones that is responsible for circumferential growth through life, is well vascularized [17]. Inhibition of angiogenesis or vasodilation during bone healing decreases the generation of new bone [18]–[21]. Interestingly, there is also a strong connection between BMP2 and vascular cells. As previously mentioned, global loss of BMP2 results in severe heart development defects [14]. Mice lacking BMP2 expression in osteoblasts and osteocytes have a reduced vascular network [15]; and recent work from our lab and others have indicated that BMP2 is expressed in vascular cells during intramembranous bone healing (stress fracture healing and distraction osteogenesis) [12], [13]. Moreover, there is a wealth of research linking arterial calcification with increased local BMP2 expression [22], [23]. To our knowledge there are no studies that have investigated the role of vascular-derived BMP2 on the skeleton.

In this study we sought to better identify the role of tissue-specific BMP2 during post-natal skeletal growth and to determine if BMP2 knockout affects the ability of terminally differentiated cells to create normal quality bone material. We targeted BMP2 knockout to two differentiated cell types known to express BMP2 during growth and healing, early-stage osteoblasts (and their progeny) and vascular endothelial cells. We then assessed bone growth, structure and strength. We hypothesized that removal of BMP2 from osteogenic and vascular cells (separately) results in smaller skeletons with inferior bone material properties.

## Methods

### Ethics Statement

This study was carried out in strict accordance with the recommendations in the Guide for the Care and Use of Laboratory Animals of the National Institutes of Health. The protocol was approved by the IACUC of Washington University of St. Louis (Protocol Number: 20110209). All efforts were made to minimize suffering. Mice were maintained in standard housing with 12 hr light/dark cycles and food and water *ad libitum.* All dual energy X-ray absorptiometry, microCT, and forelimb loading experiments were performed on live animals under anesthesia (Ketamine & Xylazine – dual energy X-ray absorptiometry or 1–3% isofluorane – microCT and forelimb loading). Euthanasia was carried out by CO_2_ asphyxiation. Three-point bending and Raman spectroscopy were performed post mortem on harvested bones.

Transgenic mice with the *Bmp2* gene floxed (obtained through materials transfer agreement with Harvard University) were crossed with either osterix-promoted Cre (Osx-Cre, B6.Cg-Tg(Sp7-tTA,tetO-EGFP/Cre)1Amc/J) [24] or vascular-endothelial-cadherin-promoted Cre (VEC-Cre, B6.Cg-Tg(Cdh5-Cre)7Mlia/J) [25]. Osx-Cre mice had a targeted deletion of BMP2 in early-stage osteoblasts, which continues as the cells develop into mature osteoblasts and osteocytes. VEC-Cre mice had a targeted deletion of BMP2 in vascular endothelial cells.

Male and female mice with the *Bmp2* floxed on one (fl/+, heterozygous) or two (fl/fl, homozygous) alleles were investigated. Throughout the paper mice with the genotype *Bmp2* ^fl/fl^; *Osx*-Cre+ are referred to as Osx-Cre cKO, while littermate mice with the genotype *Bmp2* ^fl/fl^ without Cre are referred to as Osx-Cre wildtype (WT). In addition, mice with the genotype *Bmp2* ^fl/fl^; *VEC*-Cre+ are referred to as VEC-Cre cKO, while littermate mice with the genotype *Bmp2* ^fl/fl^ without Cre are referred to as VEC-Cre WT.

### Verification of Knockout

Tissue specific knockout of BMP2 was confirmed in two ways. First, the intended cell-specific Cre expression was verified. For the Osx-Cre a green fluorescent protein (GFP) tag was already attached to the Cre protein; cells currently producing Cre appear green. The VEC-Cre was crossed into an mTmG transgenic mouse (*Gt(ROSA)26Sor^tm4(ACTB-tdTomato,−EGFP)Luo^*/J) [26]. In an mTmG mouse all normal, non-Cre expressing cells produce a red fluorescent protein (RFP) in the cell membrane, whereas cells that have expressed Cre switch to produce a GFP instead of the RFP. For both mouse lines, forelimbs (ulna and radius with surrounding muscle) were harvested (n = 3–6/genotype), fixed in 10% neutral buffered formalin overnight, decalcified in 14% EDTA, and embedded in OCT media for cryosectioning. Transverse sections were examined near the ulnar midpoint. Before imaging, the slides were rinsed in water to remove the OCT media and coverslipped. They were then imaged with 10x, 20x and 40× objective magnification (IX51, Olympus, Center Valley, PA) using TRITC and FITC filters.

Second, diminished BMP2 protein expression in the target cells was verified. For both lines, forelimbs were harvested, fixed in 10% formalin overnight, decalcified in 14% EDTA and embedded in paraffin. Transverse sections were examined near the middle of the ulna (n = 3/genotype). Immunostaining was performed using goat ABC staining system (Santa Cruz, sc-2023, Santa Cruz, CA) with BMP2 antibody (Santa Cruz, sc-6895, 1∶100 dilution overnight at 4°C) following manufacturers’ instructions. After hydrating, antigen retrieval was performed using a decloaking chamber (Biocare Medical, 95°C, 10 min, Concord, CA) containing sodium citrate buffer (pH 6.0). To visualize protein DAB was applied for 5 min. Sections were not counterstained. Imaging was performed at 40× (BX51, Olympus).

### Whole Body Measures

To measure growth body weight was recorded. *In vivo* dual energy X-ray absorptiometry (DXA, Lunar PIXImus GE) was used to measure percent fat, bone mineral content (BMC) and areal bone mineral density (aBMD) for the whole body of the mouse (excluding the head). Two separate cohorts of Osx-Cre mice were measured at 12 and 24 weeks of age (n = 4–10/group). A cohort of VEC-Cre mice were measured at 4, 8, 12, 16, 20, and 24 weeks of age (n = 4–5/group).

### MicroCT

*In vivo* microCT was performed on the left tibia of mice to determine tibia length, cortical and cancellous bone measures (VivaCT 40, Scanco Medical, Wayne, PA; X-ray tube potential 70 kV, integration time 100 ms, X-ray intensity 114 µA, isotropic voxel size 21 um, frame averaging 1, projections 500, medium resolution scan) in accordance with published guidelines [27]. The following cortical bone measures were determined for 200 µm at the tibial midpoint: total volume (TV), bone volume (BV), bone volume to total volume (BV/TV), and tissue mineral density (TMD). The following cancellous bone measures were determined for 600 µm distal to the proximal growth plate: total volume (TV), bone volume (BV), bone volume to total volume (BV/TV), volumetric bone mineral density (vBMD), trabecular number (Tb.N), trabecular connectivity density (Conn.D), structure model index (SMI), trabecular thickness (Tb.Th), and trabecular separation (Tb.Sp). Two separate cohorts of Osx-Cre mice were measured at 12 and 24 weeks of age (n = 6–9/group). A cohort of VEC-Cre mice was measured at 8, 16, and 24 weeks of age (n = 4–5/group).

### Mechanical Testing

To assess whole bone strength, mice forelimbs were loaded to failure using an established technique [28]. Briefly, mice were anesthetized and the elbow and wrist were held between two loading cups such that axial compressive loads create bending of the curved forelimb bones. The upper cup was displaced at a rate of 0.5 mm/s to compress the whole limb until complete fracture (DynaMight 8841, Instron, Norwood, MA). During displacement loading the force was recorded (LabVIEW, National Instruments, Austin, TX). Subsequent analysis identified the maximal force sustained by the whole forelimb. Two separate cohorts of Osx-Cre mice were loaded to fracture at 12 and 24 weeks old (n = 3–4/group). A cohort of VEC-Cre mice were loaded to fracture at 24 weeks old (n = 3–4/group). Mice were euthanized immediately following loading.

To assess both whole-bone strength and estimated material properties, the right and left femora of Osx-Cre mice were harvested post mortem and loaded in three-point bending (span length = 7 mm). Prior to testing, the midshaft of the femur (35 slices, 0.56 mm) was scanned using microCT (µCT40, Scanco Medical; X-ray tube potential 55 kVp, integration time 200 ms, X-ray intensity 145 µA, isotropic voxel size 16 um, frame averaging 1, projections 500, medium resolution scan). Femurs were loaded to failure at a rate of 0.1 mm/s (DynaMight 8841, Instron) while force and displacement data were collected (LabVIEW). Non-normalized properties were gathered from the force-displacement curve including stiffness, ultimate force, post-yield displacement, energy at ultimate force, and energy to fracture. Morphological parameters area and cortical thickness were calculated directly using the cross sectional microCT images. In addition, the area moment of inertia about the bending axis and the distance (c) to the farthest point of the cross section from the neutral axis were determined and used to calculate material properties of ultimate stress and Young’s modulus. Two separate cohorts of Osx-Cre mice were loaded at 12 and 24 weeks (n = 3–8/group).

### Raman Spectroscopy

Eleven bilateral humeri from six 24-week-old mice and 11 unilateral femora from eleven 12-week-old mice were used for Raman spectroscopic analysis. The 24-week-old humeri were from a mixed group of male and female mice and intended as a pilot study (see supplemental data in Tables S1). The 12-week-old femora were the same bones used for three-point bending tests so that direct correlations between strength and composition could be made. The articulating ends of the humeri were removed and the samples were centrifuged to remove marrow. After three-point-bending, the uneven fracture surface of the femora specimens were removed using an Isomet saw (Buehler, Lake Bluff, IL) and then centrifuged at 12,000 rpm to remove marrow. If organic material such as blood or bone marrow still was visible, the specimen was rinsed with PBS. If a specimen was highly fluorescent under the laser, the specimen was soaked in PBS, dried, and re-analyzed. Therefore, the Raman spectra obtained and interpreted in this study showed relatively low fluorescence. Six spectra were collected for each humerus specimen: two near the inner edge of the cortex, two in the middle of the cortex, and two near the outer edge of the cortex. Examination of the spectra from the humeri indicated that the inner edge of the cortex had higher fluorescence than the other regions, probably due to the proximity of this region to the marrow cavity, and there were no substantial differences between the right and left humeri. Thus, for each femur specimen only four spectra were collected: two in the middle of the cortex and two near the outer edge. For each parameter, medians over all spectra taken on an individual specimen were obtained and used for the statistical analysis. Four femora spectra and six humeri spectra were determined to be abnormal and were not included in the statistical analysis.

Spectra were collected using a HoloLab Series 5000 Raman microprobe (Kaiser Optical Systems Inc., Ann Arbor, MI). A laser of 532 nm excitation operated at 10 mW power (delivered to the sample surface) was focused by an 80× objective lens (N.A. = 0.75) to a beam spot of approximately 1 µm diameter. A 2048-channel CCD detector monitored signal in the spectral range 100–4000 Δcm^−1^. Each spectrum analyzed represents the average of 32 acquisitions of 4-seconds each.

Grams32^R^ (Galactic Software, Inc., Salem, NH) was used for all spectral processing. Baseline correction was performed on each spectrum. Spectral peaks were deconvolved into bands of best fit using a mixed Gaussian-Lorentzian algorithm. To assess the relative proportions of collagen and mineral, the 1003 Δcm^−1^ band was used to represent collagen and the 960 Δcm^−1 ^band was used to represent mineral [29] The width of the 960 Δcm^−1 ^band indicated the degree of crystalline organization of the mineral. To assess the degree of carbonate substitution in the mineral, the area of the 1070 Δcm^−1^ band (for Type-B carbonate substitution) was used to represent the proportion of carbonate and the area of the 960 Δ cm^−1^ band to represent the apatite mineral [30].

### Statistics

Data are presented as a mean ± standard deviation. DXA and microCT outcomes were analyzed using two-way ANOVA (factors: genotype and sex) (StatView v. 5.0, SAS Institute, Cary, NC). Individual ANOVAs were done for Osx-Cre and VEC-Cre strains, and for each timepoint. The p-values reported in the body of the text are based on these ANOVAs. If the main genotype effect was significant (p<0.05) post-hoc test Fischer’s PLSD testing was performed. In general, males had larger bones than females, and the genotype effects were similar between sexes. Furthermore, heterozygous control mice (fl/+, Cre negative) were similar to homozygous controls (fl/fl, Cre negative) in both Cre strains. Outcomes for heterozygous Cre positive mice were either similar to controls or were intermediate between the homozygous controls and homozygous knockouts. For conciseness, only data for homozygous male mice are presented in the main Tables and Figures for two timepoints (12 and 24 weeks for Osx-Cre; 16 and 24 weeks for VEC-Cre). (See the supplementary data in Tables S1 for all additional groups.) In the case of 3-point bending the right and left femur data were averaged and unpaired t-tests were performed on the pooled sets. For Raman spectroscopy differences between the two genotypes were assessed by two-sample t-tests of the spectral parameter means using Statistical Analysis System (SAS, Version 9.3 for Windows; SAS Institute, Cary, NC).

## Results

### BMP2 Deletion in Osteoblasts and Vascular Cells

**Figure 1:**
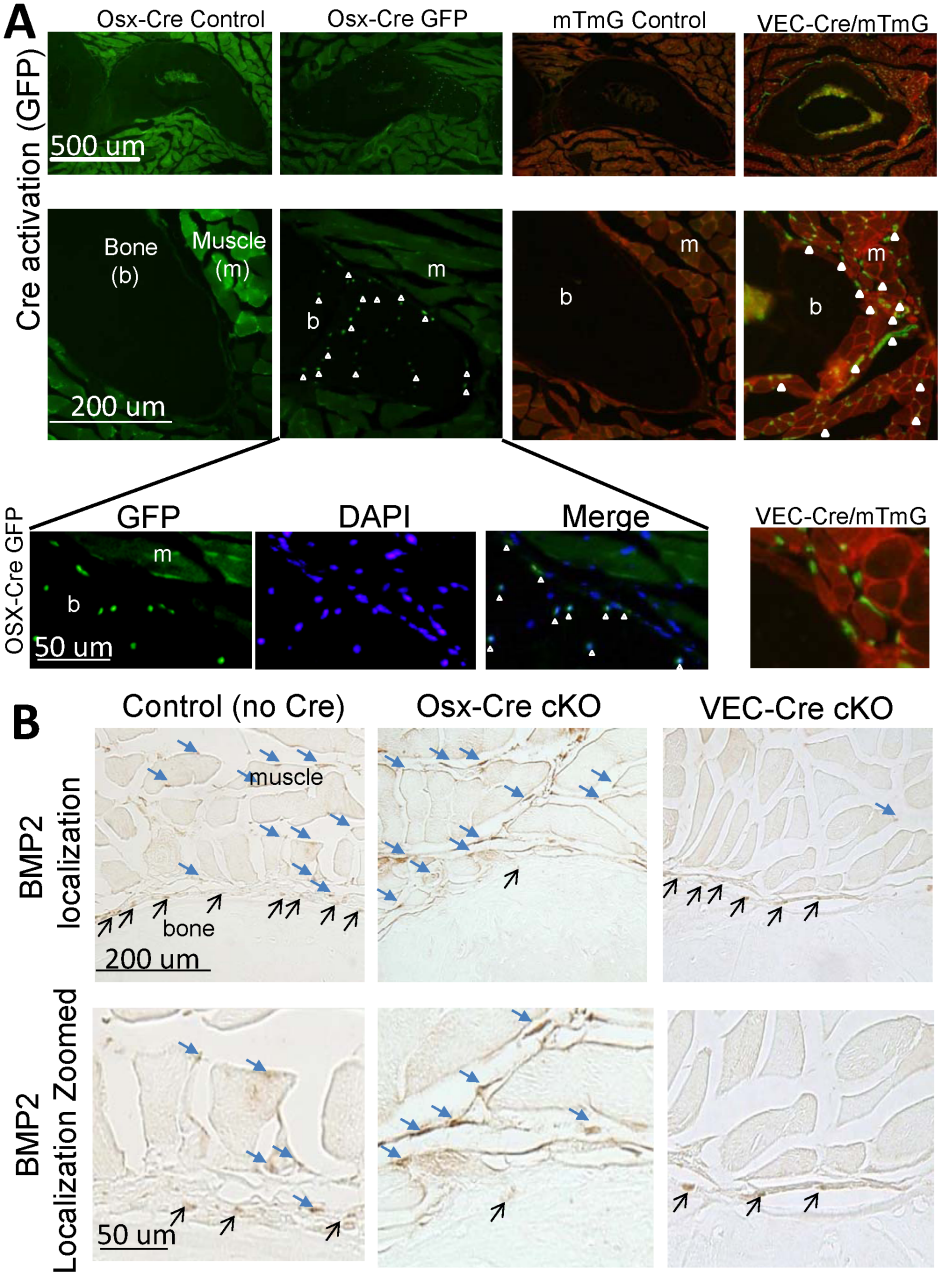
Deletion of BMP2 was evaluated in osteogenic cells (Osx-Cre) and vascular cells (VEC-Cre). (A) First, Cre activation was verified by examination of cortical bone (ulna) and muscle from transverse cross-sections through the mid-forelimb. In control samples the bone is completely black. The Osx-Cre mouse has a GFP::Cre fusion protein which demonstrated the current expression of Cre in some osteocytes within the bone and osteoblasts lining the bone surface (open white arrowheads). The VEC-Cre mouse was crossed with mTmG reporter mouse and demonstrated Cre activation within muscle and periosteum compartments surrounding the bone (filled white arrowheads). (B) Confirmation of the BMP2 protein deletion was done using immunohistochemistry. In the control sample BMP2 expression was seen in the muscle (blue filled arrows) and bone-lining cells (thin black arrows). Using the Osx-Cre BMP2 expression was absent in the bone lining cells, but abundantly expressed throughout the muscle compartment (blue filled arrows). In contrast, using the VEC-Cre expression of BMP2 was limited to osteoblasts lining the bone surface (thin black arrows). Images are representative of 3–5 sections from 3–6 animals/group.

BMP2 was successfully knocked down in our cells of interest, early stage osteoblasts and their progeny (including osteocytes), and vascular endothelial cells. The Osx- and VEC-Cres were expressed in the expected target tissues (Figure 1A, GFP signal). As seen in Figure 1A, the GFP signal of the OSX-Cre does not appear in all bone lining cells or osteocytes at any one time. This is because the GFP::Cre fusion protein is under that control of the Osterix promoter which is transiently expressed. Once the GFP::Cre protein is no longer created (i.e., Osterix is no longer being expressed) and all the GFP::Cre protein has been degraded, a cell will not fluoresce even though the knockout has occurred and the cell can no longer produce a functional BMP2 protein. The successful targeting of osteoblasts and osteocytes in post natal mice by this Osx-Cre has recently been reported [24], [31] On the other hand, in the mTmG;VEC-Cre samples, GFP highlights any cell that has ever expressed Cre (under control of the VECAD promoter). The Cre recombination causes a switch in the production of a membrane protein from RFP to GFP. The GFP expression is maintained by the constitutive activity of the Rosa locus and thus is “on” for the remainder of the cell’s life and that of any daughter cells. Immunohistochemistry showed BMP2 protein in both the muscle capillaries and bone lining cells of *Bmp2* ^fl/fl^ control mice (Figure 1B, control). BMP2 was absent in the bone lining cells of the Osx-Cre cKO mice but, as expected, remained in the muscle capillaries (Figure 1B). In contrast, BMP2 was absent in the muscle capillaries of VEC-Cre cKO mice but, as expected, remained in bone lining cells. While osteocyte expression of BMP2 was absent in all IHC samples, it is assumed that if successful BMP2 knockout in osteoblasts of the OSX-Cre line has occurred then it is also knocked out of osteocytes.

### Osteoblast Knockout of BMP2 Reduces Bone Size, but Vascular Knockout does not

**Figure 2:**
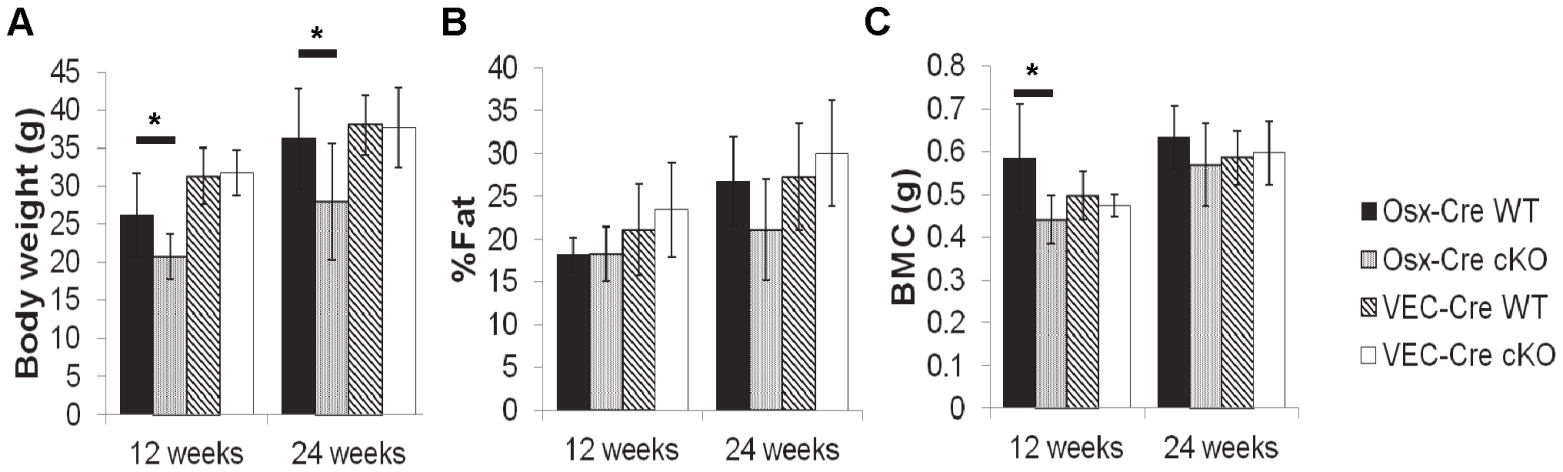
Whole body DXA results at 12 and 24 weeks for WT and cKO male mice. (A) Body weight was significantly reduced in the Osx-Cre cKO and unchanged for VEC-Cre mice. (B) Percent fat was not significantly reduced in the 12- and 24week old Osx-Cre cKO male mice (although it did reach significance at 24 weeks when male and female data were pooled (ANOVA)). (C) Osx-Cre cKO male mice showed a significant reduction in BMC at the 12-week time point, with a non-significant trend at 24 weeks (which was significant when male and female data were pooled (ANOVA)). BMC was unchanged for VEC-Cre mice at either time point.

Osx-Cre cKO mice had 20% lower body weights at both 12 and 24 weeks age (p = 0.04 and 0.004, respectively) compared to littermate (*Bmp2* ^fl/fl^) controls. In contrast, VEC*-*Cre cKO and littermate control mice had similar weights at each timepoint (Figure 2A–12 & 24 week males, Table SA in Tables S1– All groups). At 12 weeks of age the OSX-Cre cKO mice had similar percent fat as controls, but by 24 weeks old the knockouts had 6% lower percent fat (p = 0.03). VEC-Cre mice had similar percent fat as controls at each time point (Figure 2B–12 & 24 week old males, Table SA in Tables S1– All groups). DXA measurements of aBMD and BMC were lower in 12 and 24 week old Osx-Cre cKO mice versus control (p = 0.020 and 0.005, respectively, at 12 weeks; p = 0.009 and 0.015, respectively, at 24 weeks). VEC-Cre cKO had no effect on aBMD or BMC at any time point (Figure 2C–12 & 24 week males, Table SA in Tables S1– All Groups).

**Figure 3:**
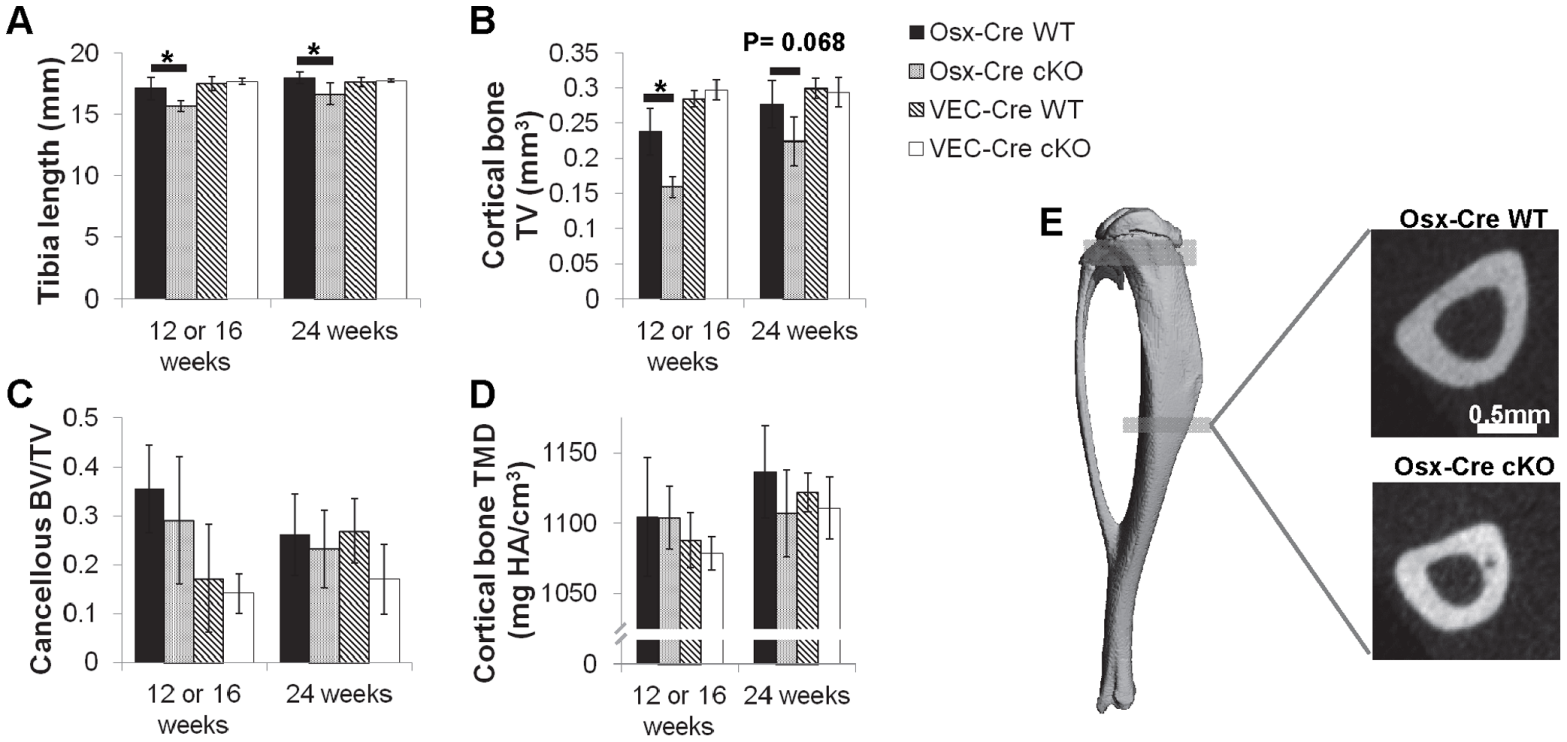
MicroCT was used to assess structural properties of the tibia at 12/16 and 24 weeks. (Osx-Cre data shown at 12 weeks, VEC-Cre data shown at 16 weeks.) Osx-Cre cKO mice had (A) shorter tibia and (B) reduced cortical bone volume with no change in (C) cancellous BV/TV or (D) cortical TMD. VEC-Cre mice showed no differences in any parameter. (E) Illustrations of cortical bone geometry show a smaller marrow cavity in the Osx-Cre cKO mice.

Tibias of Osx*-*Cre cKO mice were shorter at both 12 and 24 weeks than controls (p = 0.01 and p<0.0001, respectively, Figure 3A–12 & 24 week males, Table SB in Tables S1– All groups). Osx-Cre cKO mice at 12 weeks had smaller bones as indicated by reduced cortical TV, cortical BV, cancellous TV, and cancellous BV versus control (p = 0.006, 0.02, 0.02, 0.06, respectively; Figure 3B, 3C and Table 1 – –12 & 24 week males, Tables SB & SC in Tables S1– All groups). Interestingly, cortical BV/TV was higher indicating a relatively smaller marrow cavity (p = 0.005). Cancellous BV/TV was not affected, indicating normal spatial density of trabeculae. By 24 weeks similar differences for reduced cortical size remained in Osx-Cre cKO mice versus controls (p = 0.0005 for TV, and p = 0.017 for BV). However, cancellous BV and TV were now similar to controls. Cortical TMD of Osx-Cre cKO cortical bone was the same as littermate *Bmp2* ^fl/fl^ controls at both ages (Figure 3D). In the VEC-Cre mouse line the cKO mice had lower cortical bone BV/TV at 24 weeks (p = 0.016) and lower cancellous vBMD (p = 0.042), but in contrast to the Osx-Cre cKO, all other outcomes including those related to bone size were similar between VEC-Cre and control groups (Tables SB & SC in Tables S1– All groups).

**Table 1:** In vivo microCT results from tibia midshaft and metaphyseal regions.

Group	Genotype	Gender	Age(wk)	Numberof mice	Cortical TV(mm^3^)	CorticalBone BV/TV	Cortical BoneTMD (mg HA/cm^3^)	TrabecularTV (mm^3^)	TrabecularBV (mm^3^)	Trabecular vBMD(mg HA/cm^3^)	TrabecularThickness(mm)	TrabecularSeparation(mm)
Osx-Cre WT	BMP2 fl/fl;Osx-Cre−	Males	24	9	0.277±0.046	0.633±0.041	1140±32.4	1.43±0.145	0.377±0.149	235.9±57.7	0.071±0.008	0.189±0.040
Osx-Cre cKO	BMP2 fl/fl;Osx-Cre+	Males	24	6	0.224±0.057	0.679±0.046	1110±30.5	1.28±0.316	0.305±0.151	214.2±47.2	0.061^a^ ±0.009	0.175±0.040
Osx-Cre WT	BMP2 fl/fl;Osx-Cre−	Males	12	11	0.245±0.044	0.665±0.052	1110±37.3	1.31±0.220	0.474±0.155	305.6±76.8	0.076±0.010	0.139±0.020
Osx-Cre cKO	BMP2 fl/fl;Osx-Cre+	Males	12	6	0.159^a^ ±0.020	0.758^a^ ±0.030	1100±22.5	0.970^a^ ±0.240	0.279^a^ ±0.078	256.7±46.8	0.061^a^ ±0.006	0.138±0.030
Vec-Cre WT	BMP2 fl/fl;VEC-Cre−	Males	24	5	0.300±0.026	0.624±0.025	1120±13.8	1.47±0.163	0.388±0.075	257.7±41.3	0.071±0.006	0.187±0.030
Vec-Cre cKO	BMP2 fl/fl;VEC-Cre+	Males	24	4	0.294±0.032	0.582^z^ ±0.017	1110±21.9	1.6±0.085	0.272±0.116	191.7±53.3	0.064±0.008	0.230±0.030

z p<0.05 BMP2 fl/fl; VEC-Cre+ vs. BMP2 fl/fl; VEC-Cre−.

a p<0.05 BMP2 fl/fl; Osx-Cre+ vs. BMP2 fl/fl; Osx-Cre−.

### Osx-Cre cKO Bones have Altered Mechanical Properties at Whole-bone and Material Levels

**Figure 4:**
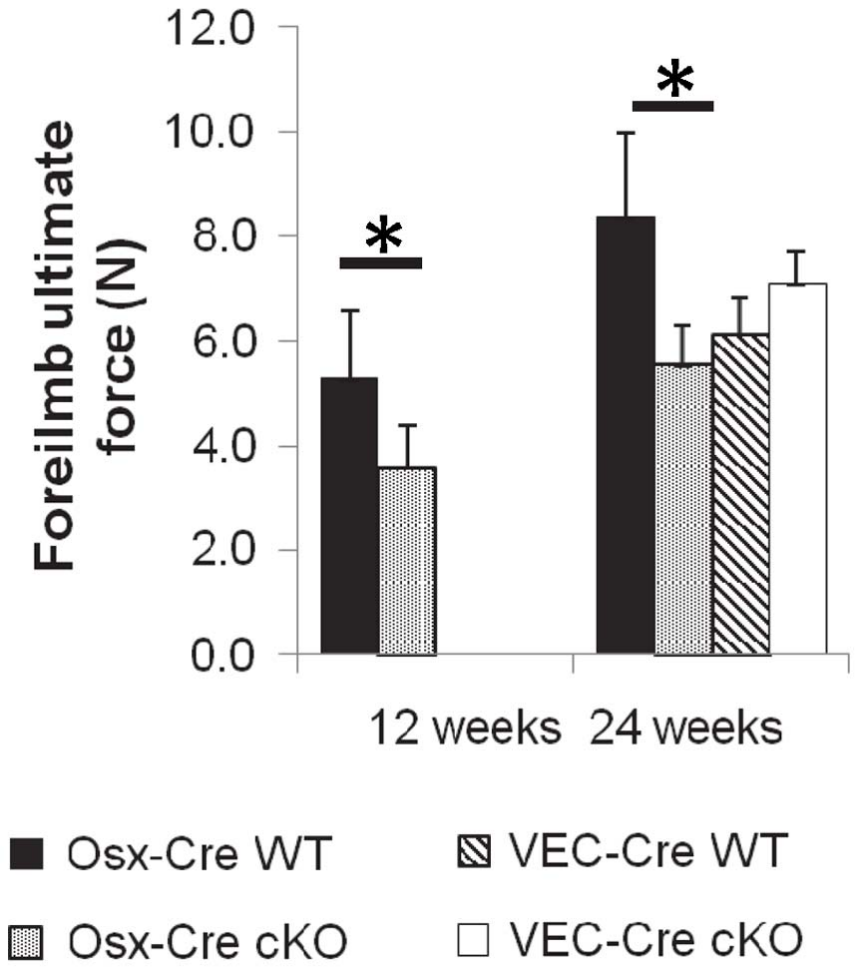
The forelimbs of mice were tested in axial compression. The ultimate force of the forelimb was significantly reduced in the Osx-Cre cKO mice at 12 and 24 weeks.

Whole forelimb strength was assessed by axial compression to failure. The ultimate force (a measure of strength of the entire structure) was significantly reduced in 12 and 24 week old Osx*-*Cre cKO mice (40% and 34%, p = 0.013 and 0.050 respectively) compared to littermate controls (Figure 4). There were no differences in ultimate force for the VEC-Cre cKO mice compared to WT.

Reduced strength of Osx*-*Cre forelimbs may be due to reduced bone size or reduced bone material properties. To assess these two factors, microCT and three-point bending were performed on femora of Osx-Cre mice. Given that the tibial structure and forelimb strength of VEC-Cre mice were not affected by BMP2 cKO, it was assumed unlikely there were any differences in the material properties and no further tests on VEC-Cre mice were performed.

**Figure 5:**
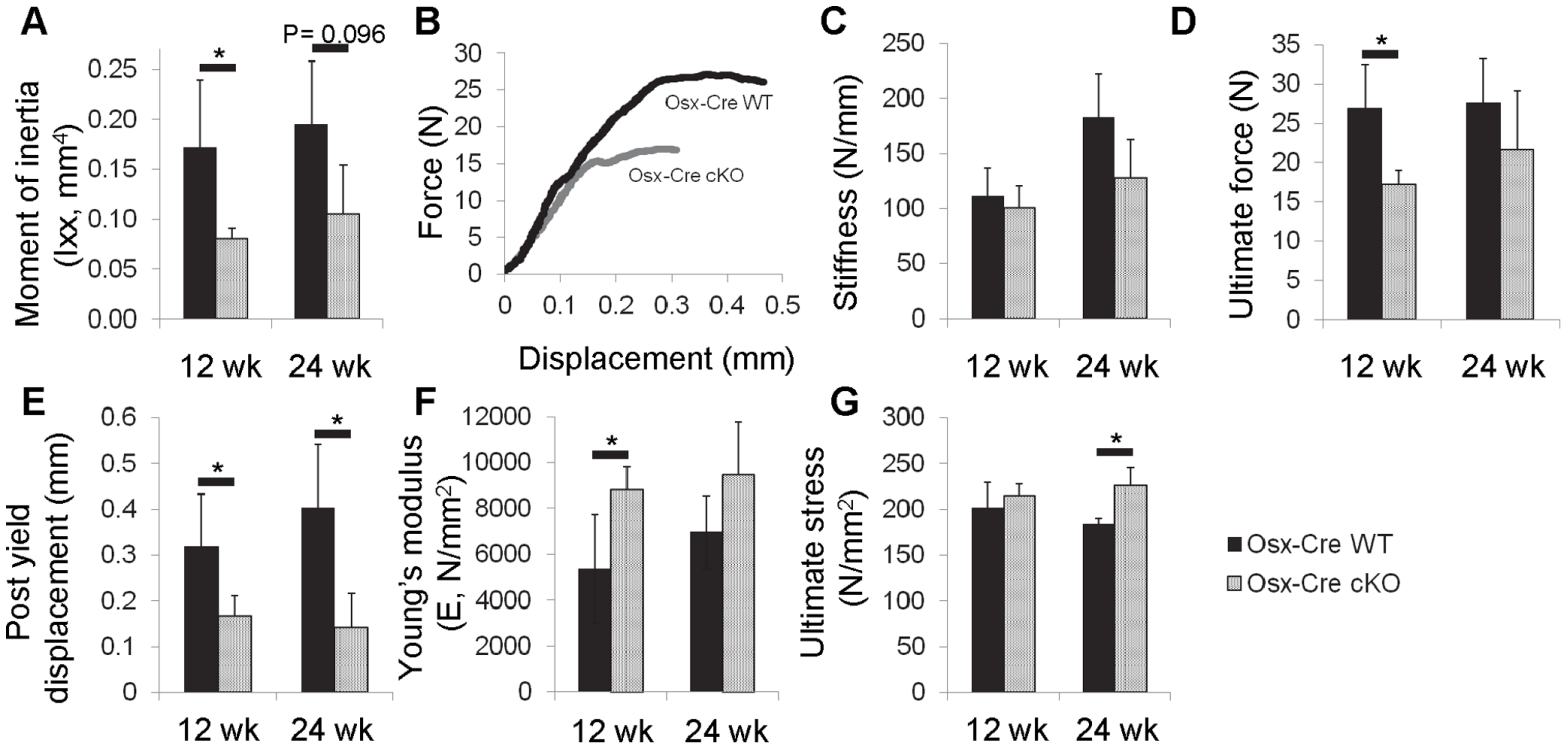
The femora of 12 and 24-Cre mice were tested in three-point bending. cKO mice had significantly reduced (A) moment of inertia compared to WT controls. (B) Example force-displacement curves for WT and cKO mice demonstrate the differences in structural properties (C) Stiffness was unchanged at either time point. However, (D) ultimate force and (E) post-yield displacement was decreased. (F) Young’s modulus for cKO mice appears to be stiffer at both time points, but is only significant at 12 wks. Conversely, (G) ultimate stress is higher in cKO but only significant at 24 weeks.

MicroCT measurements of the femur midshaft corroborated findings from the tibia, confirming a smaller bone in Osx-Cre cKO versus WT (Figure 5, Table 2). In particular, Osx-Cre cKO femora had a lower bone area, and moment of inertia (p = 0.004 and 0.006, respectively; Figure 5A). Force-displacement data from three-point bending (Figure 5B) revealed that Osx-Cre cKO femora had normal whole-bone stiffness (Figure 5C), but reduced ultimate force (p = 0.004; Figure 5D) and dramatically decreased post-yield displacement (p = 0.002; Figure 5E). In combination these properties resulted in a significantly lower energy at ultimate force and energy to fracture (p = 0.004 and 0.002, respectively, Table 2), properties that reflect overall resistance to failure. Despite the inferior whole-bone mechanical properties, femora from Osx-Cre cKO mice actually had superior stiffness and strength at the material level (i.e., after accounting for size differences), as evidenced by the increased Young’s modulus (p = 0.008; Figure 5F), and ultimate stress (p = 0.017 Figure 5G). Thus, at the material level Osx-Cre cKO bones are stiff and strong, but their reduced post-yield displacement indicates they are brittle. These material properties, when combined with their smaller size, result in a whole-bone that has normal stiffness but inferior strength and reduced energy to failure.

**Table 2:** Mechanical 3-point bending tests of mouse femora demonstrated reduced ductility in Osx-Cre cKO mice.

Group	Genotype	Gender	Age(wk)	Numbermice	Average corticalthickness (mm)	Bone Area(mm2)	Distance from centerof mass (c) (mm)	Energy at ultimateforce (N*mm)	Energy at fractureforce (N*mm)
Osx-Cre WT	BMP2 fl/fl;Osx-Cre−	Males	24	4 pairs	0.254±0.02	1.02±0.12	0.73±0.11	6.971±1.0	10.39±2.70
Osx-Cre cKO	BMP2 fl/fl;Osx-Cre+	Males	24	3 pairs	0.236±0.03	0.799±0.20	0.603±0.07	3.798±2.5	4.21^a^ ±2.85
Osx-Cre WT	BMP2 fl/fl;Osx-Cre−	Males	12	5 pairs	0.251±0.02	0.987±0.15	0.686±0.09	6.308±2.6	10.73±4.63
Osx-Cre cKO	BMP2 fl/fl;Osx-Cre+	Males	12	4 pairs	0.226±0.03	0.714^a^ ±0.06	0.575^a^ ±0.02	2.760^a^ ±0.62	3.88^a^ ±0.63
Osx-Cre 1/2 cKO	BMP2 fl/+;Osx-Cre+	Males	12	8 pairs	0.232±0.03	0.835^b^ ±0.06	0.631±0.03	4.711±1.3	7.95±3.28

a p<0.05 WT v. cKO, b p<0.05 WT v. 1/2 cKO (same ages).

*p<0.05 12wk v 24 wk (same genotype).

### Raman Spectroscopy

To determine if the differences in material properties of Osx*-*Cre bones could be due to defects in the mineral or collagen aspect of bone, we performed Raman Spectroscopy. First, a set of humeri from 24-week old mice were analyzed as a pilot study. We then analyzed the same 12-week old femora that had been used for three-point bending (Table SD in Tables S1). Carbonated hydroxylapatite was the only mineral present in both the WT and the cKO specimens. The position of the deconvolved 960 Δcm^−1^ band ranged from 960.5 to 961.3 wavenumbers for the 12-week-old femora, which is normal for bone. This range indicate some variability in the chemical components of the bone, for instance by sodium incorporation as a result of carbonate substitution [32], [33] However, the mean band positions were not statistically different between genotypes (p = 0. 92). Similarly, the 960 Δcm^−1^ bandwidth varied, indicating a small difference in atomic order, but was not statistically different by genotype (p = 0.25). The mineral-to-matrix ratio, as represented by the ratio of hydroxylapatite (area of the 960Δ cm^−1^ band) to collagen (area of the 1003 Δcm^−1^ band), was lower in the cKO mice than in WT for our pilot studies with 24-week-old humeri (p = 0.04), however the ratio did not differ by genotype for the 12-week-old femora (p = 0.37). No significant differences in features within the collagen spectral region were observed between the WT and cKO mice. To assess the degree of carbonate substitution, the area of the 1070 Δ cm^−1^ band (for Type-B carbonate substitution) was divided by the area of the 960 Δcm^−1^ band. The proportion of carbonate in the mineral varied slightly within and between specimens, as is normal in bone, but did not differ by genotype (p = 0. 84). In summary, Raman spectroscopy revealed no obvious defects in the collagen or mineral aspect of Osx-Cre cKO bones that would account for the differences in mechanical and material properties.

## Discussion

Our work shows that osteoblast-derived BMP2 is important to post-natal skeletal growth, structure and strength whereas vascular endothelial-derived BMP2 is not. Knockout of BMP2 using Osx-Cre affected both the structure and strength of long bones. Contrary to our hypothesis, the knockout of BMP2 from vascular endothelial cells did not affect any whole-body and few long-bone outcome measures for either sex, nor was the strength of the whole forelimb altered.

Structurally, Osx-Cre cKO resulted in a smaller mouse with slightly shorter limbs. This is a similar, but less severe, phenotype than when BMP2 is knocked out of all osteo- and chondro-progenitor cells using Prx1-Cre (*Bmp2* ^fl/fl^;Prx1-Cre, a.k.a, Prx1-Cre cKO) [9], [10]. The shortness of the limbs we observed may be due to the off-target knockout in non-bone cells such as hypertrophic chondrocytes [31], [34]. The Osx-Cre mouse is reported to target several non-bone cell types [31]. However, preliminary examination of growth plate morphology and cell proliferation did not identify any obvious dissimilarities that could account for the modest (10%) effect on tibial length. More importantly, the smaller bone width and reduced cortical bone volume, which would not be affected by hypertrophic chondrocytes, supports the hypothesis that BMP2 is a key factor in periosteal osteoblasts. Furthermore, the similarity of the Osx-Cre cKO phenotype to the reported phenotype in a Col 1-Cre cKO [15] suggests that the effects are from bone cell deletion of BMP2.

Periosteal cells are responsible for radial bone expansion throughout life [35] and are the main cellular contributors to fracture repair [36]. The smaller total volume of Osx-Cre cKO cortical bones indicate that if the osteoblasts and osteocytes are unable to produce BMP2, the bone forming cells in the periosteal layer fail to create enough bone to circumferentially expand similar to controls. Recent work has suggested this maybe due to indirect effects of periosteal cell BMP2 knockout. Progenitor cell behavior and vasculature architecture is altered by BMP2 knockout in early stage osteoblasts [15]. More studies are necessary to determine the relative importance of direct effects on osteoblast function versus indirect effects from other sources such as defective signals from the osteocytes or bone matrix that does not contain BMP2.

An unexpected finding is that osteoblast-derived BMP2 is involved in bone quality. The mechanical properties of Osx-Cre cKO femora reflect a combination of their reduced size coupled with size-independent properties derived from three-point bending tests on the femora which revealed that bone material from Osx-Cre cKO is stiffer (higher Young’s modulus), marginally stronger (higher ultimate stress at 24 weeks) and less ductile (decreased post-yield displacement). This means that as a material the Osx-Cre cKO bones behave just as well or better than their controls under small deformations (i.e. normal loading conditions). However, because they are less ductile, once plastic deformation occurs (i.e. trauma) the bone material fails with less energy absorbed. While not directly measured, the spontaneous fractures sustained by the Prx1-Cre cKO mice suggest a similar but more severe material defect [9], [10]. In both the Osx- and Prx1-Cre cKO lines the size discrepancy and material differences combine to create a weaker limb. Our findings are consistent with a recent report in which deletion of BMP2 using the 3.6Col1a1-Cre had a similar effect on postyield bone toughness [15].

In an initial effort to identify the source of the material property differences, we analyzed the femora with Raman spectroscopy, a technique that can characterize the structure-composition and degree of crystallinity of a material. Remarkably, there were no obvious spectroscopic differences in the mineral or collagen aspects of bone from cKO versus WT mice. Interestingly, the finding of normal bone mineral from Raman analysis of intact bones is in contradiction to the impaired in vitro mineralization in differentiated mesenchymal stem cell cultures from 3.6Col1a1-Cre; BMP2 cKO mice [15]. Nonetheless, given the strong link between bone toughness and the organic phase of bone material [37], further studies into the collagen alignment, cross linking, and structure are warranted.

A caveat to this research was off-target effects in Osx-Cre cKO teeth. All osteoblast knockout mice developed malocclusions that worsened as they aged (Figure S1). An obvious concern is that these mice could be malnourished, and the phenotype is due to diet not genetics. However, several observations argue against this possibility. First, there were no differences in percent fat in 12 week old Osx-Cre cKO mice and only modest differences at 24 weeks (Figure 2B). In mouse models of malnourishment percent fat is significantly lower (∼10%) by 12 weeks of age [38]. Furthermore, none of the heterozygous knockout mice (*Bmp2* ^fl/+^ *;*Osx-Cre+) presented malocclusions yet many of their structure and strength outcome measures fell between the knockouts and controls (see Figure S2). This shows a possible dose effect of the BMP2 knockout. Finally, a previous study by Feng *et al.* documented that this knockout not only causes malocclusions, but the mineralized tissue itself is altered; knockout teeth had extensive pitting which suggests that the teeth are brittle [39], similar to findings in our OSX-Cre cKO long bones. An additional limitation as a result of the malocclusions was that we had few Osx-Cre cKO mice at the later time point (24 weeks). Once the severity of the malocclusions was documented the experimental groups for Osx cKO were switched to a 12 week timepoint.

Knockout of BMP2 from vascular endothelial cells changed few aspects of skeletal growth up to 24 weeks of age. This was unexpected given the many links between vasculature and bone growth as well as between BMP2 and heart development, bone healing, and arterial calcification. It is possible that BMP2 from other cell types is capable of compensating for loss from vascular cells during development and maturation. Alternatively, other BMPs with overlapping functions (i.e. BMP4/7 [9]) may compensate for loss of BMP2. Further discrimination between these possibilities would require examination of BMP signaling at the cell level, which we did not evaluate here. The effects of BMP2 loss from vascular endothelial cells may only be evident in stressed situations such as a high fat diet or bone healing. It is known that BMP2 is highly elevated in vascular endothelial cells at early timepoints in healing via intramembranous bone formation (i.e. stress fracture healing and distraction osteogenesis) [13], [28]. Further studies are needed to test the requirement for vascular BMP2 in these settings.

In conclusion, the knockout of osteoblast-derived BMP2 affects the structure and strength of long bones while knockout of vascular-endothelial cell BMP2 does not. The *Bmp2* ^fl/fl^;Osx*-*Cre+ cKO mouse could serve as model to investigate the relationship between bone mass density and fracture risk associated with *Bmp2* polymorphisms in humans. The studies linking *Bmp2* polymorphisms and osteoporosis are controversial and contradictory [40]–[43]. This is due in part to the rarity of the genetic mutation, the contribution of many different factors and genes to BMD, and the relatively mild effects. Using this mouse we can investigate mechanisms that may account for increased fracture risk and then potentially translate these findings to the clinic.

## Supporting Information

Figure S1 **The teeth of Osx-Cre cKO mice were abnormal.** Example images demonstrate malocclusion in cKO mice. The teeth of ½ cKO mice were normal as were the teeth of Osx-Cre WT mice and Cre control mice (no *Bmp2* floxing).
(TIF)

Figure S2 **Results for various structural and strength measures from the Osx-Cre ½ cKO bones were between those of the Osx-Cre WT and Osx-Cre cKO.**
(TIF)

Tables S1 **Tables with DEXA (Table SA), micoCT (Tables SB and SC), and Raman Spectroscopy (Table SD) data for all experimental groups.**
(XLSX)

## References

[1] Brochmann EJ, Behnam K, Murray SS (2009) Bone morphogenetic protein-2 activity is regulated by secreted phosphoprotein-24 kd, an extracellular pseudoreceptor, the gene for which maps to a region of the human genome important for bone quality. Metabolism 58: 644–50. 19375587 10.1016/j.metabol.2009.01.001

[2] Doss MX, Gaspar JA, Winkler J, Hescheler J, Schulz H, et al (2012) Specific gene signatures and pathways in mesodermal cells and their derivatives derived from embryonic stem cells.Stem Cell Rev. 8: 43–54. 10.1007/s12015-011-9263-5 21519850

[3] Rosen V (2009) BMP2 signaling in bone development and repair. Cytokine Growth Factor Rev 20: 475–80. 19892583 10.1016/j.cytogfr.2009.10.018

[4] Lian J B, Stein GS, Javed A, van Wijnen AJ, Stein JL, et al (2006) Networks and hubs for the transcriptional control of osteoblastogenesis. Rev Endocr Metab Disord 7: 1–16. 17051438 10.1007/s11154-006-9001-5

[5] Yamaguchi A, Katagiri T, Ikeda T, Wozney JM, Rosen V, et al (1991) Recombinant human bone morphogenetic protein-2 stimulates osteoblastic maturation and inhibits myogenic differentiation in vitro. J Cell Biol 113: 681–7. 1849907 10.1083/jcb.113.3.681 PMC2288971

[6] Chandler RL, Chandler KJ, McFarland KA, Mortlock DP (2007) Bmp2 transcription in osteoblast progenitors is regulated by a distant 3′ enhancer located 156.3 kilobases from the promoter. Mol Cell Biol 27: 2934–51. 17283059 10.1128/MCB.01609-06 PMC1899916

[7] Fritz DT, Jiang S, Xu J, Rogers MB (2006) A polymorphism in a conserved posttranscriptional regulatory motif alters bone morphogenetic protein 2 (BMP2) RNA:protein interactions. Mol Endocrinol 20: 1574–86. 16497730 10.1210/me.2005-0469

[8] Wohl GR, Towler DA, Silva MJ (2009) Stress fracture healing: fatigue loading of the rat ulna induces upregulation in expression of osteogenic and angiogenic genes that mimic the intramembranous portion of fracture repair. Bone 44: 320–30. 18950737 10.1016/j.bone.2008.09.010 PMC2759644

[9] Bandyopadhyay A, Tsuji K, Cox K, Harfe BD, Rosen V, et al (2006) Genetic analysis of the roles of BMP2, BMP4, and BMP7 in limb patterning and skeletogenesis PLoS Genet. 2: e216. 10.1371/journal.pgen.0020216 PMC1713256 17194222

[10] Tsuji K, Bandyopadhyay A, Harfe BD, Cox K, Kakar S, et al (2006) BMP2 activity, although dispensable for bone formation, is required for the initiation of fracture healing. Nat Genet 38: 1424–9. 17099713 10.1038/ng1916

[11] Wu L, Feng J, Wang L, Mu Y, Baker A, et al (2011) Development and characterization of a mouse floxed Bmp2 osteoblast cell line that retains osteoblast genotype and phenotype. Cell Tissue Res 343: 545–58. 21271257 10.1007/s00441-010-1120-3 PMC3050048

[12] McKenzie JA, Silva MJ (2011) Comparing histological, vascular and molecular responses associated with woven and lamellar bone formation induced by mechanical loading in the rat ulna. Bone 48: 250–8. 20849995 10.1016/j.bone.2010.09.005 PMC3021598

[13] Matsubara H, Hogan DE, Morgan EF, Mortlock DP, Einhorn TA, et al (2012) Vascular tissues are a primary source of BMP2 expression during bone formation induced by distraction osteogenesis. Bone 51: 168–80. 22391215 10.1016/j.bone.2012.02.017 PMC3719967

[14] Zhang H, Bradley A (1996) Mice deficient for BMP2 are nonviable and have defects in amnion/chorion and cardiac development. Development 122: 2977–86. 8898212 10.1242/dev.122.10.2977

[15] Yang W, Guo D, Harris MA, Cui Y, Gluhak-Heinrich J, et al (2013) Bmp2 gene in osteoblasts of periosteum and trabecular bone links bone formation to vascularization and mesenchymal stem cells. J Cell Sci 126: 4085–98. 23843612 10.1242/jcs.118596 PMC3772385

[16] Kronenberg HM (2003) Developmental regulation of the growth plate. Nature 423: 332–6. 12748651 10.1038/nature01657

[17] Simpson AH (1985) The blood supply of the periosteum. J Anat 140: 697–704. 4077705 PMC1165093

[18] Tomlinson RE, McKenzie JA, Schmieder AH, Wohl GR, Lanza GM, et al (2013) Angiogenesis is required for stress fracture healing in rats. Bone 52: 212–9. 23044046 10.1016/j.bone.2012.09.035 PMC3513671

[19] Tomlinson RE, McKenzie JA, Schmieder AH, Wohl GR, Lanza GM, et al.. (2013) Nitric Oxide Mediated Vasodilation Increases Blood Flow During the Early Stages of Stress Fracture Healing. J App Physio. EPub December 19, 2013. 10.1152/japplphysiol.00957.2013 PMC3921351 24356518

[20] Jacobsen KA, Al-Aql ZS, Wan C, Fitch JL, Stapleton SN, et al (2008) Bone formation during distraction osteogenesis is dependent on both VEGFR1 and VEGFR2 signaling. J Bone Miner Res 23: 596–609. 18433297 10.1359/JBMR.080103 PMC2674537

[21] Hausman MR, Schaffler MB, Majeska RJ (2001) Prevention of fracture healing in rats by an inhibitor of angiogenesis. Bone 29: 560–4. 11728927 10.1016/s8756-3282(01)00608-1

[22] Hruska KA, Mathew S, Saab G (2005) Bone morphogenetic proteins in vascular calcification. Circ Res 97: 105–14. 16037577 10.1161/01.RES.00000175571.53833.6c

[23] Li X, Yang HY, Giachelli CM (2008) BMP-2 promotes phosphate uptake, phenotypic modulation, and calcification of human vascular smooth muscle cells. Atherosclerosis 199: 271–7. 18179800 10.1016/j.atherosclerosis.2007.11.031 PMC3249145

[24] Rodda SJ, McMahon AP (2006) Distinct roles for Hedgehog and canonical Wnt signaling in specification, differentiation and maintenance of osteoblast progenitors. Development 133: 3231–44. 16854976 10.1242/dev.02480

[25] Alva JA, Zovein AC, Monvoisin A, Murphy T, Salazar A, et al (2006) VE-Cadherin-Cre-recombinase transgenic mouse: a tool for lineage analysis and gene deletion in endothelial cells. Dev Dyn 235: 759–67. 16450386 10.1002/dvdy.20643

[26] Muzumdar MD, Tasic B, Miyamichi K, Li L, Luo L (2007) A global double-fluorescent Cre reporter mouse. Genesis 45: 593–605. 17868096 10.1002/dvg.20335

[27] Bouxsein ML, Boyd SK, Christiansen BA, Guldberg RE, Jepsen KJ, et al (2010) Guidelines for assessment of bone microstructure in rodents using micro-computed tomography. J Bone Miner Res 25: 1468–86. 20533309 10.1002/jbmr.141

[28] Martinez MD, Schmid GJ, McKenzie JA, Ornitz DM, Silva MJ (2010) Healing of non-displaced fractures produced by fatigue loading of the mouse ulna. Bone 46: 1604–12. 20215063 10.1016/j.bone.2010.02.030 PMC2875275

[29] Wopenka B, Kent A, Pasteris JD, Yoon Y, Thomopoulos S (2008) The tendon-to-bone transition of the rotator cuff: a preliminary Raman spectroscopic study documenting the gradual mineralization across the insertion in rat tissue samples. Appl Spectrosc 62: 1285–94. 19094386 10.1366/000370208786822179 PMC2701203

[30] Penel G, Leroy G, Rey C, Bres E (1998) MicroRaman spectral study of the PO4 and CO3 vibrational modes in synthetic and biological apatites. Calcif Tissue Int 63: 475–81. 9817941 10.1007/s002239900561

[31] Chen J, Shi Y, Regan J, Karuppaiah K, Ornitz DM, et al (2014) Osx-Cre targets multiple cell types besides osteoblast lineage in postnatal mice. PLoS One 9: e85161. 24454809 10.1371/journal.pone.0085161 PMC3893188

[32] Skinner HCW (2005) Biominerals. Mineral Mag 69: 621–641.

[33] Li Z, Pasteris JD (2014) Chemistry of bone mineral, based on teh hypermineralized rostrum of the beaked whale Mesoplodon densirostris. Am Mineral : In Press. 10.2138/am.2014.4571 PMC4251810 25484370

[34] Oh JH, Park S, de Crombrugghe B, Kim JE (2012) Chondrocyte-specific ablation of Osterix leads to impaired endochondral ossification. Biochem Biophys Res Commun 418: 634–40. 22290230 10.1016/j.bbrc.2012.01.064 PMC4012832

[35] Seeman E (2007) The periosteum–a surface for all seasons. Osteoporos Int 18: 123–8. 17180552 10.1007/s00198-006-0296-6

[36] Colnot C, Zhang X, Knothe Tate ML (2012) Current insights on the regenerative potential of the periosteum: molecular, cellular, and endogenous engineering approaches. J. Orthop. Res. 30(12): 1869–78. 10.1002/jor.22181 PMC4620732 22778049

[37] Nyman JS, Makowski AJ (2012) The contribution of the extracellular matrix to the fracture resistance of bone. Curr Osteoporos Rep 10: 169–77. 22527725 10.1007/s11914-012-0101-8 PMC7980275

[38] Devlin MJ, Cloutier AM, Thomas NA, Panus DA, Lotinun S, et al (2010) Caloric restriction leads to high marrow adiposity and low bone mass in growing mice. J Bone Miner Res 25: 2078–88. 20229598 10.1002/jbmr.82 PMC3127399

[39] Feng J, Yang G, Yuan G, Gluhak-Heinrich J, Yang W, et al (2011) Abnormalities in the enamel in bmp2-deficient mice. Cells Tissues Organs 194: 216–21. 21597270 10.1159/000324644 PMC3178081

[40] Medici M, van Meurs JB, Rivadeneira F, Zhao H, Arp PP, et al (2006) BMP-2 gene polymorphisms and osteoporosis: the Rotterdam Study. J Bone Miner Res 21: 845–54. 16753015 10.1359/jbmr.060306

[41] McGuigan FE, Larzenius E, Callreus M, Gerdhem P, Luthman H, et al (2007) Variation in the BMP2 gene: bone mineral density and ultrasound in young adult and elderly women. Calcif Tissue Int 81: 254–62. 17726567 10.1007/s00223-007-9054-9

[42] Styrkarsdottir U, Cazier JB, Kong A, Rolfsson O, Larsen H, et al (2003) Linkage of osteoporosis to chromosome 20p12 and association to BMP2. PLoS Biol 1: E69. 14691541 10.1371/journal.pbio.0000069 PMC270020

[43] Varanasi SS, Tuck SP, Mastana SS, Dennison E, Cooper C, et al (2011) Lack of association of bone morphogenetic protein 2 gene haplotypes with bone mineral density, bone loss, or risk of fractures in men. J Osteoporos 2011: 243465. 22013543 10.4061/2011/243465 PMC3195445

