# Supplementary figures and images for: Long Bone Structure and Strength Depend on BMP2 from Osteoblasts and Osteocytes, but Not Vascular Endothelial Cells
Source: PLoS One. 2014 May 16;9(5):e96862. doi: 10.1371/journal.pone.0096862 (PMC4024030; doi:10.1371/journal.pone.0096862)

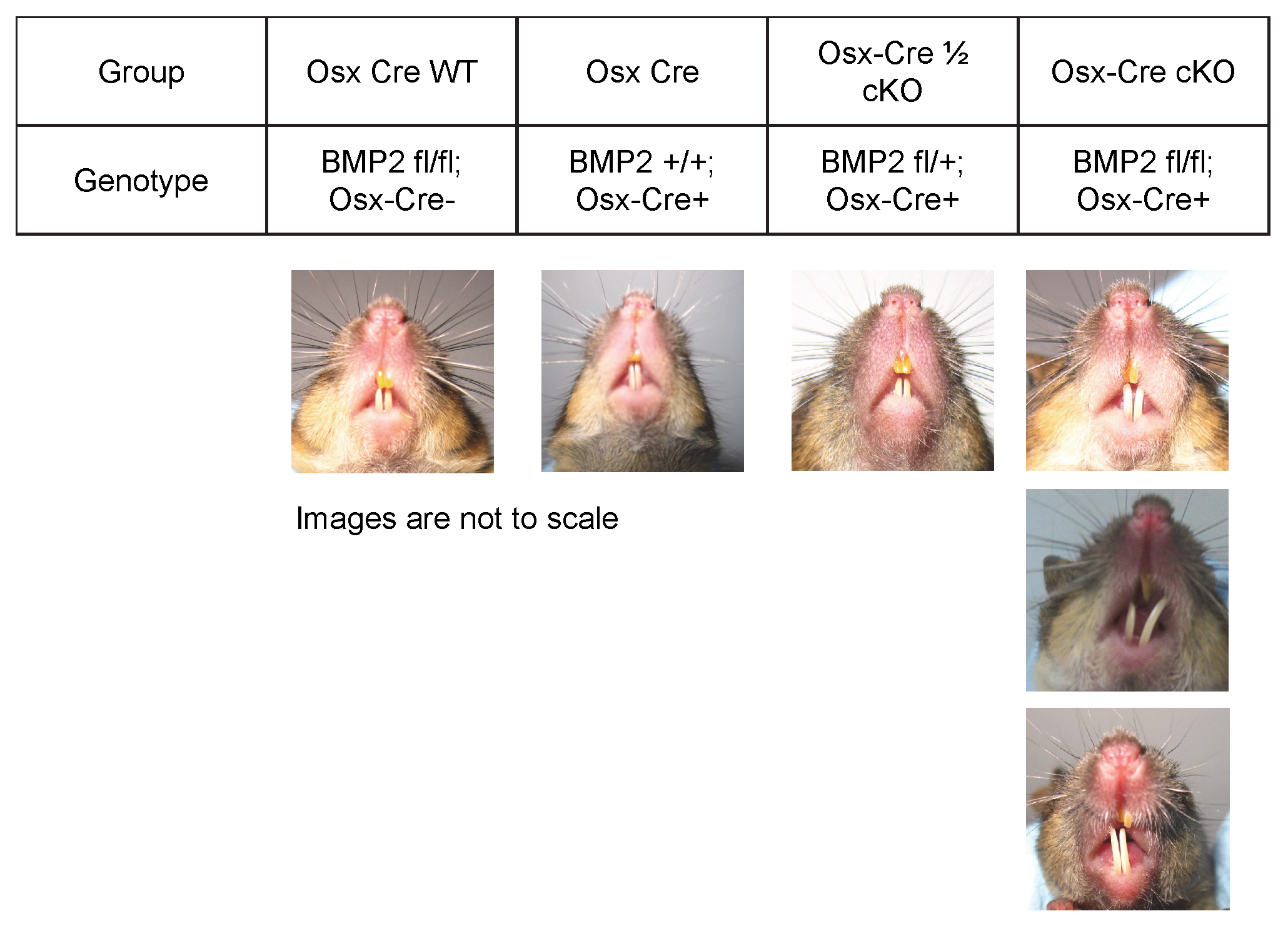

Supplement: Figure S1 — The teeth of Osx-Cre cKO mice were abnormal. Example images demonstrate malocclusion in cKO mice. The teeth of ½ cKO mice were normal as were the teeth of Osx-Cre WT mice and Cre control mice (no Bmp2 floxing). (TIF) [file pone.0096862.s001.tif]

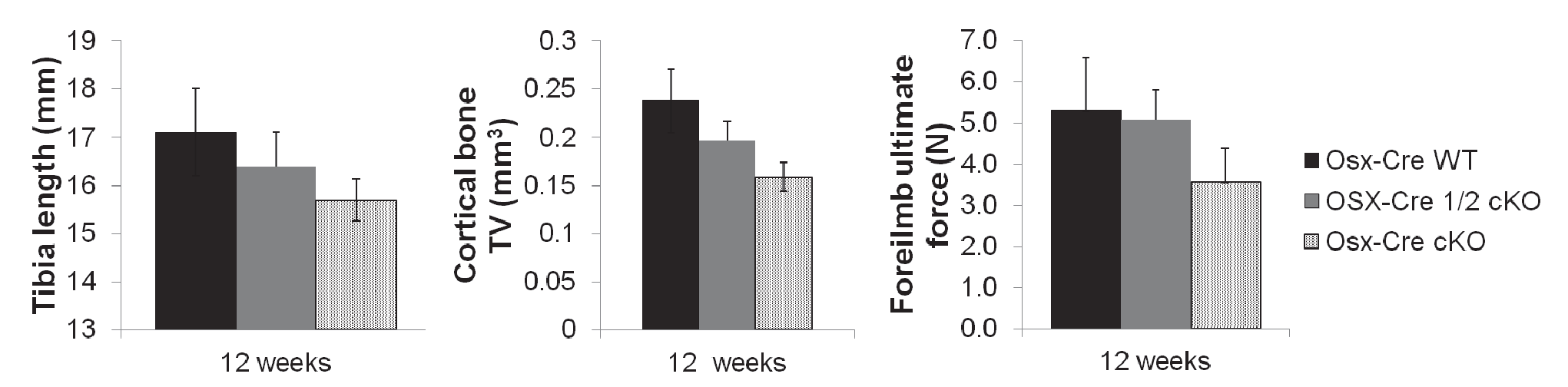

Supplement: Figure S2 — Results for various structural and strength measures from the Osx-Cre ½ cKO bones were between those of the Osx-Cre WT and Osx-Cre cKO. (TIF) [file pone.0096862.s002.tif]
